# Cognitive Remediation as a Tool for Enhancing Treatment Dimensions of Schizophrenic Symptomatology: A Systematic Review of Randomized Controlled Trials

**DOI:** 10.3390/brainsci15101130

**Published:** 2025-10-21

**Authors:** Maria Skokou, Panagiotis-Diogenis Stavridis, Aikaterini Ntoskou-Messini, Lambros Messinis

**Affiliations:** 1 Laboratory of Neuropsychology and Behavioral Neurosciences, Department of Psychology, Aristotle University of Thessaloniki, University Campus, 54124 Thessaloniki, Greece; panagiwthsstav@gmail.com (P.-D.S.); lmessinis@auth.gr (L.M.); 2 Independent Researcher, 26221 Patras, Greece; katerinaalogotherapy@gmail.com; 3 Department of Psychiatry, University of Patras Medical School, 26504 Patras, Greece

**Keywords:** psychosis, schizophrenia, cognitive, negative, positive, depression, rehabilitation

## Abstract

**Background/Objectives**: Despite efforts, schizophrenia remains a difficult disease to treat for cognitive, positive, negative, and mood symptoms. In the present review, we explore existing data on the ameliorating effects of neurocognitive rehabilitation and the diverse symptomatology of the disorder. **Methods**: This systematic review has been registered with PROSPERO (registration number: CRD 420251154674). Following PRISMA guidelines, we conducted a search in PubMed, Scopus, and Science Direct database from inception to 14 July 2025. The methodological quality assessment was made by applying the Joanna Briggs Institute (JBI) critical appraisal tool for systematic reviews. **Results**: Of the 1001 records screened for eligibility, thirty-five studies were identified for data extraction and synthesis. Of these, seven had a low risk of bias, and seven had a high bias risk. The effects of cognitive remediation on the symptoms of schizophrenia were varied. There are consistently positive effects on negative symptoms, but the findings are mixed regarding other domains of symptomatology. The therapeutic effect on positive psychotic symptoms correlated with the severity of symptoms at baseline. Efficacy for mood and anxiety symptoms is controversial, with a comparable number of studies yielding contradicting results. **Conclusions**: Cognitive remediation has been shown to represent a significant therapeutic tool for schizophrenia symptoms. The method‘s efficacy seems well-established for negative symptoms, whereas the effects on positive psychotic, mood, and anxiety symptoms, although promising, are currently mixed. More high-quality research targeting patient populations where the symptoms studied are more prominent is needed to clarify the effectiveness of the intervention for distinct dimensions of schizophrenic symptomatology.

## 1. Introduction

Schizophrenia is a devastating disorder, imposing a heavy burden on patients, families, and society [1]. The introduction of antipsychotic agents brought forth a revolution in the treatment of the disease, succeeding in bringing many patients living in asylums back to the community [2]. Still, achieving recovery or even symptomatic remission remains elusive to this day, and even when remission is achieved, many patients experience residual or persisting symptoms, cognitive decline, and functional failure [3]. Cognitive deficits are a core characteristic of schizophrenia and notably resistant to treatment. They include deficits in working and verbal memory, processing speed, attention, executive function, and reasoning, among others [4]. Deficits in social cognition are also apparent and highly detrimental; they seriously interfere with the ability of schizophrenic patients to communicate effectively and achieve or maintain social communication [5]. Medication, including antipsychotics as well as novel agents (d-cycloserine, memantine, and anticholinesterase inhibitors represent some examples), have proven ineffective or minimally effective [6]. However, besides cognitive symptoms, other symptom domains also tend to persist and often show resistance to available pharmaceutical agents, creating serious distress in patients’ lives and even frustration for families and treating clinicians [7].

These symptoms include positive symptoms, such as delusions and hallucinations, negative symptoms, such as abulia, loss of interest, blunted affect, withdrawal, and anergia, mood symptoms, most usually depressive, and others. The latter cause serious distress, frustration, and suicidal thoughts or attempts in patients [8]. The situation is grim, and novel or additional therapies that can increase the therapeutic effect and outcome are more than welcome; their implementation is crucial and even necessary.

Cognitive remediation (CR) is a rehabilitative therapeutic procedure that takes advantage of the brain’s neuroplastic ability, essentially to increase the patient’s cognitive capacity and functional outcome. It has been practiced in a broad variety of medical conditions, beginning with traumatic brain injury, but it was quickly implemented in psychiatric disorders, including schizophrenia, for the treatment of cognitive deficits that are almost universally present in such patients [9].

Due to the important benefits of CR, this method has been included by the European Psychiatric Association in the treatment guidelines for cognitive deficits of schizophrenia [10]. Recent studies have shown a positive effect of CR on additional clinical symptoms, and it possibly represents an indispensable therapeutic modality for the management of patients with schizophrenia. The present review is a comprehensive overview of the efficacy of CR in treating clinical symptoms beyond the positive effects of medication and assists in increasing familiarity and confidence among clinicians for the benefit of their patients.

## 2. Materials and Methods

### Literature Search

A comprehensive search was conducted in PubMed, Scopus, and Science Direct databases from inception to 14 July 2025. We used the following search string for all three databases screened for eligible articles: ((cognitive remediation) OR (cognitive rehabilitation) OR (cognitive training)) AND psychopathology OR delusions OR hallucinations OR negative OR depressive OR anxiety). Across databases, the search fields were title, abstract, and keywords. A search of the gray literature was not performed. Reference lists of relevant papers were searched manually for additional studies. Our aim was to provide data derived from original research. The following inclusion criteria were applied based on the PICO framework for systematic reviews. (1) Population: Samples consisting of patients with schizophrenia, diagnosed based on specific classification or operationalized criteria. (2) Intervention: An experimental group that underwent cognitive remediation to target specific cognitive deficits, whether computerized, paper-and-pencil, or otherwise. (3) Comparator: An RCT design where a control group and randomized allocation were utilized. The control group interventions included various treatments, such as pharmacotherapy, psychotherapy, or occupational therapy (a complete list of the control group interventions is presented in Table 1). (4) Outcome: Changes in cognitive and clinical symptoms assessed using relevant scales and explicitly reported. Exclusion criteria were as follows: (1) study designs or article types other than original, i.e., case reports, conference proceedings, reviews, meta-analyses, study protocols, (2) study designs other than randomized controlled trials, (3) articles published in languages other than English, and (4) not specified or unclear reporting of cognitive and clinical outcomes. Articles were excluded at the full-text stage if (1) samples consisted of patients with multiple disorders from the schizophrenia spectrum within a group, including schizoaffective disorder, (2) the control group was unsuitable as a comparator, mainly because of a cognitive remediation intervention being implemented, or (3) there was no mention of the preferred method of diagnosis confirmation (e.g., DSM-V).

**Figure 1:**
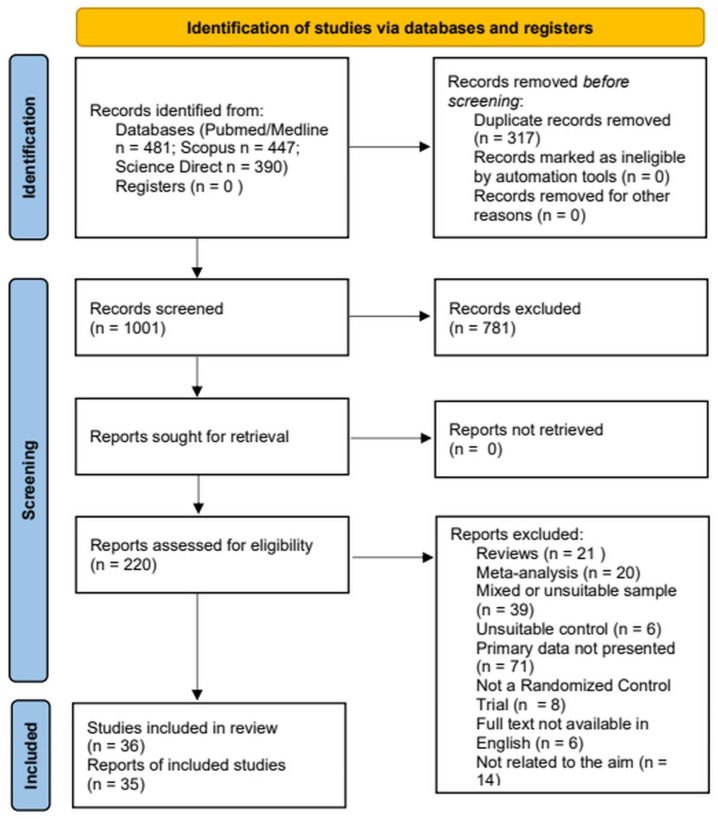
PRISMA 2020 flow diagram for the systematic review, which included searches of PubMed/Medline, Science Direct, and Scopus electronic databases.

This systematic review is registered with the International Prospective Register of Systematic Reviews (PROSPERO; registration number: CRD 420251154674). The review was conducted according to the PRISMA guidelines for reporting systematic reviews [11]. Abstract and full-text screening was conducted by two independent reviewers (MS and PS) using the Cadima evidence synthesis tool and database [12]; disagreement was resolved by consensus or by consulting with the supervisor of the study (LM). The flow diagram of the screening and selection of studies is presented in Figure 1.

Data from included studies were extracted and organized into tables summarizing study characteristics, interventions, outcomes, and risk of bias. Supplementary Table S3 presents the effect size indices used—η^2^, ηp^2^, d, ES, total b—in a comprehensive manner to facilitate understanding. The methodological quality of the studies was assessed using the revised JBI critical appraisal tool for the risk of bias of RCTs [13] (Supplementary Tables S1 and S2). Each study was evaluated across 13 criteria covering different sources of methodological bias. All included records were assessed independently by two reviewers (MS and PS); disagreement was resolved through consensus. A composite rating for every study was calculated as follows: studies meeting >70% of the criteria were rated “low” risk, those meeting 50–70% were rated “moderate” risk, and studies meeting <50% were rated as having a “high” risk of bias. A detailed breakdown of each study’s criterion rating is provided in Supplementary Table S4.

No attempts were made to retrieve missing data from the study authors. We considered missing information to reflect the authors’ reporting decisions; therefore, only the data available in the published reports were extracted.

## 3. Results

### 3.1. Overview

After database searches and screening, a total of 35 studies were included in the analysis (Table 1). Most studies were of moderate methodological quality; however, seven of them had low risk of bias [14,15,16,17,18,19,20]. Sample sizes ranged between 11 [21] and 270 [17], with the majority recruiting less than 100 patients. Interventions were diverse, including computerized or paper-and-pencil training, individually or in groups, virtual reality, and social cognition or metacognition components, whereas control groups received Treatment as Usual (TAU) with medication only or various types of psychosocial therapies, such as supportive therapy, music and dancing therapy, occupational rehabilitation, and others. Most studies explored dimensions of psychopathology by applying the Positive and Negative Symptoms Scale (PANSS), while some focused on depressive symptoms or anxiety. About one-third [17,21,22,23,24,25,26,27,28,29,30,31,32] examined clinical effects as a secondary outcome. Of the studies, 13 reported measures of effect sizes [16,22,25,28,30,33,34,35,36,37,38,39,40].

### 3.2. Specific Symptom Outcomes

Positive symptoms were assessed with the use of the PANSS (positive subscale) or, rarely, the Brief Psychiatric Rating Scale (BPRS), the Present State Examination (PSE), or the Scale for the Assessment of Positive Symptoms (SAPS). Of the studies that conducted these assessments (*n* = 34), only four [22,36,41,42] demonstrated a significantly superior effect of cognitive remediation over TAU or other rehabilitation (art therapy, physical training, occupational therapies) [41]. Only one [41] reported an effect size, which was found to be small. The sample of two studies consisted of inpatients, acutely ill patients [22], or patients in rehabilitation centers [41]. Duration of treatment was 4 to 24 weeks.

**Table 1:** Studies assessing changes in clinical symptoms following cognitive rehabilitation.

Study	Country	Sample (Training/Control, Age, % Males, Setting)	Type of Intervention	Study Duration and Dose	Cognitive Functions Targeted	Control Group	Results	Risk of Bias
Gharaeipour and Scott, 2012 [43]	Iran	*N* = 42, 21/21 CR 29.81 ± 7.61 GST 27.62 ± 5.66 71.4%. Inpatients, consecutive admissions	Cognitive remediation	1 h/session, 6 sessions/w, 40 h, 8 weeks	Attention, working memory, executive functions	Group supportive therapy	No significant differences in PANSS, BDI, BAI	Moderate
Zhu et al., 2021 [22]	China	*N* = 72, 22/24/26 CCT: 30 (25–39) CCT + MSST: 32 (26–41) Controls: 33 (26–41) 51.3% Inpatients, acute admissions, outpatients	Compensatory Cognitive Training combined with Medication Self-Management Skills Training; Compensatory Cognitive Training	CCT: 2 h/session, 2 sessions/w, 4 weeks CCT + MSST: CCT: 2 h/session, 2 sessions/w MSST: 2 h/ session, 3 sessions/w, 4 weeks	Prospective memory, conversational attention, task attention, verbal learning and memory, cognitive flexibility, problem solving, planning	Treatment as Usual (psychopharmacological therapy)	Significant group × time interaction for CCT + MSST compared with TAU in PANSS positive symptoms (MD = −0.101, *p* < 0.03, η^2^ = 0.211, 95% CI [−0.178, −0.025]) No significant difference between CCT + MSST and CCT or CCT and TAU	Moderate
Vita et al., 2011b [41]	Italy	*N* = 31, 16/15 IPT-cog: 34.6 (7.6) Treatment as Usual: 39.9 (8.6) 83.8% Inpatients from rehabilitation center	Integrated Psychological Therapy (IPT)	45 min/session, 2 sessions/w, 24 weeks	Attention (selective and sustained), memory, conceptualization abilities, cognitive flexibility	Treatment as Usual (psychopharmacological therapy)	Significant difference on PANSS total score (IPT: 95.8 ± 14.4 → 66.7 ± 11.5; control: 94.0 ± 11.7 → 77.8 ± 14.9, *p* < 0.04) Significant difference in PANSS negative scores (IPT: 31.3 ± 6.4 → 21.6 ± 5.6; control: 28.8 ± 6.8 → 26.0 ± 8.4, < 0.001)	Moderate
Penadés et al., 2006 [15]	Spain	*n* = 60, 20/20/20 CRT 34.43 (8.3) CBT 35.84 (8.5) Treatment as Usual 38.30 (9.1) 63% Outpatients, chronic	Cognitive remediation therapy	1 h/session, 2–3 sessions/w, 16 weeks, 40 sessions	Flexibility in thinking and information set maintenance, executive processes central to memory control, working memory, planning	CBT, Treatment as Usual (psychotropic medication)	Significant difference for the PANSS depression items (CBT: 10.9 ± 3.0 → 6.6 ± 1.9; CRT: 10.3 ± 3.3 → 116 ± 3.4); no significant differences for other subscales	Low
Vita et al., 2011a [16]	Italy	*N* = 84, 26/30/28 IPT-Cog: 37.15 ± 9.10 Cogpack: 36.87 ± 11.40 REHAB: 43.00 ± 7.76 69% Inpatients from rehabilitation centers	Integrated Psychological Therapy–Cognitive Remediation; Cogpack	45 min/session, 2 sessions/w, 24 weeks	IPT-Cog: cognitive differentiation, social perception, verbal communication, social skills, and interpersonal problem solving; Cogpack: verbal memory, verbal fluency, psychomotor speed and coordination, executive function, working memory, attention, language and calculation skills	Rehabilitation: art therapy, physical training, or occupational therapies	Significant differences between groups in CGI-S (IPT-Cog: −0.96 ± 0.72, ES = −80; Cogpack: −0.80 ± 0.76, ES = −0.57; rehabilitation: −0.36 ± 0.78, *p* = 0.02) Significant differences between groups for PANSS positive scores (IPT-Cog: 5.0 ± 3.40; ES = −0.83; Cogpack: −5.47 ± 4.92; ES = −0.79; rehabilitation: −1.79 ± 4.33, *p*< 0.001) Significant differences between groups for PANSS negative scores (IPT-Cog: −6.96 ± 5.6, ES = −1.29; Cogpack: −4.90 ± 6.31, ES = −0.94; rehabilitation: 0.04 ± 4.13, *p* < 0.001); significant differences between groups for PANSS total scores (IPT-Cog: −21.65 ± 15.40, ES = −1.19; Cogpack: −20.80 ± 18.35, ES = −1.03; rehabilitation: −3.71 ± 14.68, *p* < 0.001)	Low
Zhu et al., 2022 [17]	China	*N* = 270, 144/72/54 CCRT 46.60 ± 8.94 CRT 47.56 ± 8.23 Active control 46.11 ± 8.21 63.7%	Computerized cognitive remediation therapy; cognitive remediation therapy	45 min/session, 4–5 sessions/w, 12 weeks, 50 sessions	Cognitive flexibility, working memory and planning, facial emotion recognition, context emotion estimation, and emotional management	Active control: dance learning, playing a simple instrument	No significant effects on PANSS scores	Low
Zhu et al., 2020 [23]	China	*N* = 157, 78/79 CCRT 43.74 (9.24) Treatment as Usual 43.65 (8.64) 54.1% Community-dwelling, clinically stable	Computerized cognitive remediation therapy	45 min/session, 4–5 sessions/w, 12 weeks	Cognitive flexibility, working memory, planning, social functions, i.e., emotion management	Treatment as Usual (medication)	No significant effects on PANSS scores	Moderate
Tan et al., 2016 [18]	China	*N* = 90, 44/46 CRT: 46.77 ± 7.18 MDT: 46.09 ± 5.52 60% Inpatients, chronic	Group cognitive remediation therapy, Frontal/Executive Function Program (Revised) (Chinese)	1 h/session, 4 sessions/w, 10 weeks, 40 sessions	Flexibility in thinking and information set maintenance, working memory, goal-oriented, set/schema formation, manipulation, and planning	Musical and Dancing Therapy (MDT)	No significant effect on PANSS scores	Low
D’Amato et al., 2011 [44]	France	*N* = 77, 39/38 Intervention 33.4 ± 6.9 Control 32.2 ± 6.0 75.3% Outpatients, remitted	Cognitive remediation therapy, Rehacom	2 h/session, 2 sessions/w, 7 weeks, 14 sessions	Attention/ concentration, working memory, logic, and executive functions	Treatment as Usual, waiting list	No significant effect on PANSS, CGI scores	High
Ricarte et al., 2012 [33]	Spain	*N* = 50, 24/26 Active 38.34 ± 9.6 Control 35.21 ± 13.3 82% Inpatients, outpatients	Event-Specific Memory Training	90 min/session, 1 session/w, 10 weeks	Autobiographical memory	Social skills and occupational therapy	Significant differences between groups for BDI scores (experiment: 18.25 ± 12.3 → 10.16 ± 8.4; control: 12.42 ± 10.2 → 11.92 ± 11.0, *p* = 0.006, ηp^2^ =0.15)	Moderate
Omiya et al., 2016 [20]	Japan	*N* = 17, 8/9 Fep 43.25 ± 14.50 Control 39.00 ± 11.09 41.1% Inpatients, outpatients, chronic	Frontal/Executive Program	60 min/session, 2 sessions/w, 24 weeks, 44 sessions	Cognitive flexibility, working memory, planning	Treatment as Usual (psychopharmacological therapy)	Significant differences for PANSS total scores (FEP: 79.9 ± 7.9 → 68 ± 10.2; control 77.4 ± 6.2 → 78.1 ± 7.59, *p* < 0.03)	Low
Wykes et al., 2007 [24]	U.K.	*N* = 40, 21/19 CRT 18.8 (2.6) Control 17.5 (2.2) 65% inpatients at follow-up	Cognitive remediation therapy	1 h/session, 3 sessions/w, 12 weeks	Memory, cognitive flexibility, planning	Treatment as Usual (psychopharmacological therapy)	No significant effect on SPRS	Moderate
Rakitzi et al., 2016 [25]		*N* = 48, 24/24 IPT 31.3 ± 7.2 Control 33.8 ± 6.7 66% Outpatients	Integrated Psychological Therapy (IPT) – Group Therapy Cognitive Component	1 h/session, 2 sessions/w, 10 weeks, 20 sessions	Vigilance/attention, working memory, verbal memory, social perception	Treatment as Usual (psychopharmacological)	Significant differences between groups for PANSS negative scores (IPT: 33.5 ± 4.5 → 26.1 ± 4.3 → 24.0 ± 4.6; control: 31.0 ± 4.3 → 30.3 ± 5.8 → 28.9 ± 4.7; T1–T2: *p* = 0.00 d = 0.89; T1–T3: *p* = 0.00 d = 1.12); significant differences between groups for PANSS total scores (IPT: 59.9 ± 14.3 → 45.6 ± 9.4 → 43.9 ± 13.8; control: 59.0 ± 12.6 → 55.5 ± 9.9 → 52.2 ± 13.0; T1–T3: *p* = 0.01, d = 0.75)	Moderate
Wykes et al., 2003 [26]	U.K.	*N* = 33, 17/16 CRT 36.5 (19–55) Control 40.6 (24–64) 75% Outpatients	Cognitive remediation therapy	12 weeks	Flexibility, memory, planning	Intensive occupational therapy activities	No significant effect on BPRS scores	Moderate
Sachs et al., 2012 [34]	Austria	*N* = 38, 20/18 TAR 27.20 ± 7.17 Treatment as Usual 31.72 ± 9.35 52.6% Inpatients, outpatients	Training of Affect Recognition (TAR)	2 sessions/w, 6 weeks, 12 sessions	Facial affect recognition	Treatment as Usual, occupational therapy	Significant within-group differences for PANSS negative scores (TAR: 27.35 ± 7.72 → 18.45 ± 6.18, *p* < 0.001 d = 1.27); significant interaction between group and time (F(1,36) = 12.671, *p* = 0.001); significant within-group differences for BDI scores (TAR 13.00 ± 9.82 → 8.25 ± 8.16. d = 0.53, *p* = 0.001)	High
Giuliani et al., 2024 [45]	Italy	*N* = 40, 20/20 Intervention 37.15 ± 9.96 Control 36.70 ± 9.44 70% Outpatients	Modified Social Cognition Individualized Activities Lab (mSoCIAL)	30 min/session, 1 session/w, 10 weeks	Social cognition and metacognitive skills, emotion recognition, Theory of Mind, narrative enhancement	Treatment as Usual (pharmacological, psychological, rehabilitative, occupational)	No significant effect on PANSS scores	High
Li et al., 2022 [35]	China	*N* = 62, 30/32 VRT 46 (37, 50) Control 47.5 (37.25, 51.75) Gender Males 62.9% Inpatients, remitted	Virtual reality (VR)	5 sessions/w, 2 weeks	Working memory, processing speed, attention, verbal memory, visual memory, reasoning problem solving, social cognition	Treatment as Usual (antipsychotic treatment)	Significant pre and post differences for PANSS general scores (VR: 19 (18, 23) vs. 17 (16, 21)); Treatment as Usual 19 (17.5, 21) vs. 19 (17.25, 20.75), *p* = 0.016, ES = 0.458 Significant difference for volition scores, VR < Treatment as Usual, *p* = 0.014	Moderate
Fathi et al., 2025 [19]	Iran	*N* = 54, 27/27 CCT41.78 ± 5.22 Control 40.67 ± 8.04 61%	Computerized Cognitive Training (CCT)-CANTAB	1 h/session, 3 sessions/w, 10 weeks	Spatial Recognition Memory (SRM), Paired Associate Learning (PAL), Spatial Working Memory (SWM), Spatial Planning and Spatial Span (SSP)	Active control: computer games with high cognitive demands	Significant main effects of time and time × group interaction on DASS-D scores (CCT: MD = −1.85, 95% CI [−1.90, 0.42], *p* = 0.005); significant time × group interaction for DASS-S scores within the CCT group; T1 and T2 were significantly higher than T0 (MD (95% CI) = 2.96 (1.54 to 4.38), *p* < 0.001; MD = 2.67, 95% CI [1.18, 4.16], *p* < 0.001); significant difference between the two groups at both T1 and T2 (MD = 2.15, 95% CI [0.69, 3.61], *p* < 0.001; MD = −1.93, 95% CI [3.27, −0.59], *p* < 0.001); significant difference between the intervention and control groups (MD (95% CI) = 4.30 (1.38 to 7.22), *p* < 0.001), with the positive effects of the intervention persisting up to 3 months post-intervention (MD (95% CI) = −3.70 (−6.54 to −1.18), *p* = 0.001)	Low
Zhang et al., 2024 [46]	China	*N* = 40, 20/20 CCRT 48.200 ± 2.114 Control 46.850 ± 2.048 100% Inpatients, institutionalized	Computerized cognitive remediation therapy	40 min/session, 5 sessions/w, 8 weeks	Attention, working memory, speed of processing, cognitive flexibility, reasoning and problem solving, social cognition	Treatment as Usual (medication only)	Significant time × group interaction for PANSS total scores (CCRT: 77.30 ± 2.68 vs. 75.90 ± 2.72 vs. 74.90 ± 2.85; control: 80.90 ± 2.11 vs. 80.90 ± 2.11 vs. 80.90 ± 2.11, *p* < 0.001); significant within-group differences for PANSS negative scores (CCRT: 27.00 ± 1.21 vs. 26.15 ± 1.21 vs. 25.65 ± 1.24; control: 27.40 ± 1.27 vs. 27.40 ± 1.27 vs. 27.40 ± 1.27, *p* < 0.001); significant time × group interaction for HDRS (CCRT: 5.25 ± 0.68 vs. 3.20 ± 0.56 vs. 2.75 ± 0.43; control: 4.10 ± 0.56 vs. 3.35 ± 0.64 vs. 4.40 ± 0.91, *p* < 0.015)	High
Dai et al., 2022 [27]	China	*N* = 82, 25/26/31 CAE 41.50 (8.72) Aerobic 41.40 (7.86) Control 44.06 (8.40) 75.6% Inpatients, remitted	Computerized cognitive remediation therapy; CCRT + aerobic exercise = CAE	30 min/session, 2 sessions/w, 8 weeks	Processing speed, cognitive flexibility	Aerobic, Treatment as Usual (antipsychotics, psychological consultation, medical care, and behavior modification)	Significant pre and post differences for PANSS negative scores (CAE: −2.69 (1.83); AE: −1.48 (2.22), control: −1.06 (2.37), CAE vs. control: *p* = 0.018 ES = 0.096)	Moderate
Fekete et al., 2022 [36]	Hungary	*N* = 46, 23/23 MCT 44.22 ± 10.45 Control 38.39 ± 10.41 47.8% Outpatients	Group Metacognitive Training (MCT)	1 session/w, 16 weeks	Mental flexibility, jumping to conclusions, emotion recognition, Theory Of Mind, metacognitive functioning, attributional style	Treatment as Usual (psychopharmacological therapy, regular psychiatric control and care)	PANSS between groups post intervention positive, b = −4.66, *p* = 0.045, disorganized b = −5.98, *p* = 0.018, total b = −14.34, *p* = 0.026; between groups, post vs. 6 months positive b = −4.78 *p* = 0.046, disorganized b = −6.89 *p* = 0.022, total b = −14.95, *p* = 0.033 Within-group, mct post vs. baseline positive total b = −10.44, *p* = 0.029, negative b = −3.84, *p* = 0.048, disorganized b = −4.57 *p* = 0.007, post vs. 6 months no difference Control: no difference Baseline PANSS scores (≥75 PANSS total score or <75 PANSS, greater improvement) T0–T1 B = −21.8 *p* < 0.001 T0–T2 B = −16.9, *p* = 0.046	Moderate
Sampedro et al., 2021 [28]	Spain	*N* = 94, 47/47 Rehacop 40.60 ± 10.45 Control 41.43 ± 10.41 83% Inpatients, outpatients, rehabilitation unit	Rehacop + psychoeducation	60 min/session, 3 sessions/w, 20 weeks	Attention, visual and verbal learning, recall, recognition memory, working memory; language: verbal comprehension, verbal fluency, and abstract language; executive functions planning, problem solving, cognitive flexibility, reasoning, categorization and conceptualization, processing speed, social cognition emotion	Active control, occupational group activities (gardening, sewing, handicrafts, painting, and music), psychoeducation	Significant pre and post differences for negative scores (Rehacop: 6.83 [−9.18, −4.58]; control: −1.60 [−3.60, −0.12], *p* = 0.03, ηp^2^ = 0.108) Disorganization Rehacop: −0.97 [−1.48, −0.49]; control: −0.13 [−0.47, −0.25], *p* = 0.007, ηp^2^ = 0.086 Excitement Rehacop: −1.20 [−1.74, −0.65]; control: −0.24 [−0.98, −0.49], *p* = 0.041, ηp^2^ = 0.049	Moderate
Rocha et al., 2021 [21]	Portugal	*N* = 11, 6/5 SCIT 29.5 ± 13.38 Control 27 ± 6.12 90.9% Outpatients, illness duration <2 years	Group Social Cognition and Interaction Training (SCIT)	45–60 min/ session, 1 session/w, 20 sessions	Theory of Mind, emotion perception, attributional bias	Psychoeducation	No significant effect on PSP or PANSS scores	Moderate
Bossert et al., 2020 [37]	Germany	*N* = 59, 19/18/21 I-CACR 32.37 ± 8.71 G-CACR 28.68 ± 9.43 Treatment as Usual 29.67 ± 6.65 72.8% Inpatients, outpatients	Group Computer-Assisted Cognitive Remediation CogniPlus (I-CACR); Individualized Computer-Assisted Cognitive Remediation (I-CACR)	50 min/session, 4 sessions/w, 5 weeks	Attention: alertness, selective, divided; working memory, executive functions	Treatment as Usual (pharmacological and psychotherapeutic treatment, occupational therapy, and social skill training)	No significant effect on PANSS or HAMD scores, except for self-reported depression (BDI-scores), where a main effect of time was revealed: F(1, 52) = 22.82, *p* < 0.001, ηp^2^ = 0.31 for the total sample	Moderate
Matsuda et al., 2018 [29]	Japan	*N* = 62, 31/31 Outpatients	Japanese Cognitive Rehabilitation Program for Schizophrenia (JCORES)	60 min/session, 2 sessions/w, 12 weeks	Attention, psychomotor speed, learning, memory, executive functions	Treatment as Usual, waiting list	Significant differences between groups for PANSS on PANSS general subscales (JCORES: −3.17 ± 4.33; control: −0.06 ± 5.93, *p* = 0.032)	Moderate
Peña et al., 2016 [30]	Spain	*N* = 101, 52/49 Inpatients, outpatients	Rehacop Social Cognitive Intervention and Functional Skills Training	90 min/session, 3 sessions/w, 16 weeks	Attention: sustained, selective, alternating, divided; memory: visual and verbal learning, recall, recognition; language: verbal fluency, verbal comprehension, abstract language; executive functions: planning, social cognition	Occupational group activities	Significant effect on PANSS negative scores (Rehacop: −5.29 [−6.45, −4.13]; control: −2.82 [−4.01, −1.62] *p* = 0.004, ηp^2^ = 0.082) Emotional distress (Rehacop: −2.68 [−3.33, −2.02]; control: −0.81 [−1.49, −0.14], *p* = 0.001, ηp^2^ = 0.136); negative subfactor (Rehacop −5.29 [−6.45, −4.13]; control: −2.82 [−4.01, −1.62], ES = 0.082); differences for negative subdomains: social amotivation, *p* = 0.005, ηp^2^ = 0.077	Moderate
Cella et al., 2014 [38]	U.K.	*N* = 85 Community mental health teams	Cognitive rehabilitation	3 sessions/w, 40 sessions	Executive functions, working memory, long-term memory, attention	Treatment as Usual (psychopharmacological therapy)	Significant reduction of negative symptoms and disorganization in the CR group: W_Neg, F(2, 80) = 21.1, *p* < 0.0001, ηp^2^ = 0.07, and W_Dis, F(2, 80) = 14.2, *p* < 0.0001, ηp^2^ = 0.1	Moderate
Klingberg et al., 2011 [14]	Germany	198 36.9 ± 9.9 56.1%	Cognitive rehabilitation, restitution, compensation of cognitive deficits	47.5 min/session (mean), 13.7 sessions	Attention, memory, executive functions	CBT	No significant effect on PANSS, SANS, CDSS, or CGI scores	Low
Kayser et al., 2006 [31]	France	14 Video: 32.4 ± 9.4 Control: 38.2 ± 9.2 50%	Theory of Mind Training using videos depicting emotional interactions	1 session/w, 12 weeks	Theory of Mind	Treatment as Usual (psychopharmacological therapy)	No significant effects on PANSS and BPRS scores	High
Reeder et al., 2004 [32]	U.K.	31 31.3 ± 13.5 16–64 73%	Cognitive rehabilitation training	3 sessions/w, 40 sessions	Attention, memory; executive functions: cognitive shift, working memory, planning	Occupational therapy, activities to account for therapist contact	No significant effects on BPRS scores	High
Beigi et al., 2008 [42]	Iran	42 Unclear Unclear	Cognitive rehabilitation therapy	30–45 min/session, 2 sessions/w, 8 weeks	Attention, memory, executive function, abstract thinking	Treatment as Usual (pharmacological therapy)	Significant differences between groups for SAPS scores (CRT: 66.15 ± 13.98 → 53.75 ± 12.32, TAU: 66.85 ± 15.21 → 66.35 ± 17.71, *p* < 0.001); significant differences between groups for SANS scores (CRT: 59.35 ± 13.05 → 54.1 ± 12.37; TAU: 63.15 ± 10.29 → 63.85 ± 10.83, *p* < 0.05)	High
Tao et al., 2015 [47]	China	86 CR: 28.95 ± 7.38 Control: 29.71 ± 6.36 54.6%	Cognitive rehabilitation	30 min/session, 2 sessions/w, 16 weeks	Memory, attention, language, executive functions, coordination	Treatment as Usual, pharmacological	No significant effects on PANSS scores	
Yamanushi et al., 2024 [39]	Japan	15/15	Cognitive remediation therapy, Rehacom	60 min/session, 2 sessions/w, 12 weeks, 24 sessions	Attention/vigilance, working memory, verbal learning and memory, visual learning and memory, reasoning and problem solving, and social cognition	Treatment as Usual (psychopharmacological therapy)	Significant time × group interaction for (SANS) anhedonia/asociality scores (Rehacom: 21.57 ± 4.16 → 18.36 ± 4.80; control: 21.69 ± 3.84 → 21.23 ± 3.30, *p* = 0.019, ES = 0.19); no significant effect on the PANSS and other subscales	Moderate
Ojeda et al., 2012 [48]	Spain	93 33.81± 9.7/37.75± 8.3 81.1%	Rehacop	90 min/session, 3 sessions/w, 12 weeks	Attention, processing speed, memory, language, executive functions, social cognition	Occupational therapy	Significant difference between groups when controlling cognitive change in insight and CGI scores improved (Rehacop-insight: 5.46 ± 3.5 → 7.92 ± 3.1; control: 8.50 ± 4.4 → 8.64 ± 4.2) (Rehacop-CGI: 5.12 ± 1.3 → 4.12 ± 1.3; control CGI: 4.63 ± 1.3 → 3.94 ± 1.5)	High
Sánchez et al., 2014 [40]	Spain	N = 92, 36/48 Rehacop: 33.60 ± 9.4 Control: 36.92 + 10.5 69.5%	Rehacop	90 min/session, 3 sessions/w, 12 weeks	Attention, memory, processing speed, language, executive functions, social cognition	Treatment as Usual (psychopharmacological therapy)	Significant time × group interaction between groups for negative symptoms (Rehacop: 27.23 ± 11.6 → 21.91 ± 9.4; control: 24.85 ± 9.7 → 22.84 ± 10.1, ES = 0.48); significant time × group interaction for disorganization scores (Rehacop: 17.03 ± 7.2 → 12.91 ± 5.6; control 14.13 ± 5.4 → 12.67 → 6.1, ES = 0.58) Significant time × group interaction for emotional distress (Rehacop: 10.97 ± 6.2 → 7.66 ± 3.9; control: 7.95 ± 4.7 → 6.33 ± 3.5, ES = 0.47); significant differences for PANSS total scores (Rehacop: 99.39 ± 34.8 → 74.83 ± 23.5; control: 84.56 ± 25.1 → 71.70 ± 25.6, ES = −0.50)	Moderate

On the contrary, statistically significant improvements in negative symptoms were more readily found and are reported by 12 studies. Of those, five report effect sizes, large [25,27,28] or medium to large [30,38], in samples including outpatients, remitted inpatients, or both. Specifically, Peña et al. and Yamanushi et al. reported improvements in social amotivation and anhedonia/asociality with medium to large effect sizes [30,39]. Duration of studies was between 6 and 24 weeks.

Five studies also report significant superiority of cognitive remediation interventions vs. control conditions on the general PANSS subscale, with small, medium, or large effect sizes [25,29,35,41,46], and another five for total PANSS scores [16,20,36,41,46]. In the study by Penadés et al. [15], which compared cognitive remediation vs. CBT in a sample of chronic outpatients, CBT outperformed cognitive remediation in the general PANSS subscale, while CR was superior in the cognitive factor of PANSS. Positive results are also reported for disorganization [28,36,38,40], with large sizes [28], for excitement, with medium sizes [30,40], and for emotional distress, with a large effect size. Fekete et al. investigated whether baseline symptom severity had any impact on the degree of symptomatic improvement; they found that a PANSS score of >75 was predictive of greater improvements of symptoms following cognitive rehabilitation [36].

Insight was explored by Ojeda et al. [48], where a significantly superior effect over occupational therapy was reported. Volition was found to respond better to CR vs. TAU, according to Li et al. [35].

Nine studies focused on other symptoms of schizophrenia. Regarding depression, significantly superior results were reported by five studies [19,33,34,37,46], with a small to moderate effect size. Two studies yielded negative outcomes [14,43]. In the study by Penadés et al. [15], CBT proved superior to CR for the treatment of depressive symptoms. As for anxiety, the study by Fathi et al. [19] found better performance of CR on the stress subscale of DASS (DASS-S], but not anxiety. Also, Gharaeipour and Scott [43] obtained negative results regarding anxiety.

Only two studies reported long-term follow-up assessments at 3 or 6 months and showed that therapeutic effects seem to persist [19,36].

## 4. Discussion

Although cognitive remediation has well-documented effects on cognitive deficits of patients with schizophrenia, results for other symptoms seem promising, yet not unequivocally. In the present review, the existing evidence is mixed, with positive and negative results for various aspects of psychopathology. There are several reasons that could lead to this inconsistency. A significant portion of the studies included in the present systematic review had one or multiple methodological issues. Information regarding blinding, group allocation, or inter–rater reliability was often omitted from the studies. Furthermore, the exclusion of articles in other languages instead of English could have excluded relevant findings. Patient samples are mostly rather small, and studies present significant heterogeneity in terms of setting, including inpatient, outpatient in the community or in rehabilitation, and day centers, phase of illness, whether acute, chronic, remitted, or early course, type and components of intervention, dosing, including number of sessions and duration of treatment, and control conditions, including Treatment as Usual or treatments other than cognitive remediation. Although a meta-analysis could possibly clarify or reduce the confusion, the degree of heterogeneity would compromise the comparability of the studies and the feasibility of meta-analytic processing. It is expected that effects on patients with different characteristics would vary. It is probable that in situations where positive symptoms are severe, there is a larger deviation of scale scores than in a remitted condition and a larger range for putative improvement, which is more promptly demonstrable by the statistical analyses. This could be the case for the positive results of the studies on acutely ill patients, contrary to most studies, which yield negative results. In stable subjects, on the other hand, marked negative symptoms with minimal scores on positive subscales are the most common presentation; hence, it is for negative symptoms that patients would experience more benefits. In the same line stand the findings of Fekete et al. [36], where total scores of PANSS > 75 are associated with significantly greater improvements from the intervention. Given that a large percentage of patients are only partially remitted or refractory despite adequate trials of antipsychotic medication, the therapeutic potential of cognitive remediation becomes particularly important.

Although the effectiveness of the intervention cannot be definitively ascertained at this point, given that effect sizes of the improvements were in many cases large or medium and follow-up assessments demonstrated maintenance of the therapeutic benefits, this is very important for the substantiation of the efficacy of cognitive rehabilitation methods. Furthermore, investigation of the neurobiological processes that underly the therapeutic effects and mediate the transfer of the cognitive gains to other areas of clinical state and functioning remains crucial. Some studies have investigated neurobiological and neuroimaging aspects of cognitive rehabilitation that could be relevant to this matter. Increased activation and functional connectivity after cognitive remediation have been reported in multiple regions, such as the prefrontal cortex and thalamic regions [49,50]. Sampedro et al. [51], based on the sample of patients they examined in their previous study [28] (which is included in the present review), found greater cortical thickness in the right temporal lobe (right temporal pole, inferior temporal gyrus, middle temporal gyrus, superior temporal gyrus, and fusiform gyrus) at post-treatment compared to pre-treatment in the REHACOP group but not in the active control group. These are areas especially pertinent to the pathophysiology of positive and negative schizophrenic symptoms. More specifically, dysfunction in temporal areas has been associated with auditory hallucinations and disruption of fronto-striato-thalamic circuits with delusions [52,53]. Negative symptoms are thought to be related to the hypofunction of frontal areas, including the anterior cingulate cortex [54]. Frontal lobe dysfunction has been identified as a neural correlate of depression [55]. Furthermore, enhanced self-esteem is related to the newly achieved functional gains, which adds to the patients’ positive affect [56].

A groundbreaking line of research focuses on the glymphatic system’s function in relation to psychosis. This system is a pathway based on the astrocytes, and it allows for the exchange of cerebrospinal fluid with interstitial fluid through a paravascular network formed by the astrocytes, with aquaporin-4 (AQP4) having a central role. Normally, this leads to the removal of solute metabolic waste from the cerebrospinal fluid to the lymphatic system and thus to a clean and stable brain microenvironment [57,58]. Intact sleep, particularly delta-rich slow-wave (N3) sleep, is crucial for optimal system function [59].

It has been recently indicated that people with psychosis, even at an early stage of illness, show compromised function of the glymphatic system compared to healthy controls [60], and such a dysfunction is correlated with cognitive impairment [61] and psychotic symptoms [62].

Could this action work both ways? More specifically, could the amelioration of cognitive functioning through cognitive remediation help normalize glymphatic function? Although currently this is just a hypothesis, there are some supporting recent findings. Enhanced cognitive function seems to improve resting-state functional connectivity [63] and relieve sleep problems [64], which could result in enhancing glymphatic system function. This, in turn, would allow for more efficient removal of metabolic waste, a stable brain microenvironment, and improvement of the status of neuroglia and astrocytes, with further empowerment of large-scale brain circuits. Because of these changes, psychotic and cognitive symptoms might be further reduced, establishing a virtuous circle of clinical improvement.

In view of the above, many issues remain to be elucidated. The preceding neurobiological explanation of the therapeutic effects of cognitive remediation, although aligned with current knowledge on brain function, remains only hypothetical and fragmentary, calling for robust, extensive research. Questions regarding optimal elements of the intervention and the duration, dosing, and timing of the therapy must be answered. Documentation of the cost-effectiveness of the method is another critical issue that would facilitate the broader delivery of cognitive rehabilitation. To this end, there is a need for more studies with low risk of bias and larger samples of patients. Standardized outcome measures and long-term follow-up would further strengthen evidence around the effectiveness and the persistence of the therapeutic effects. The present data indicate that a careful recommendation of this intervention can be offered alongside treatment as usual while awaiting more definitive data.

## 5. Limitations

Limitations of the present study include the fact that the literature search was conducted using three databases only. This might have led to the omission of studies available through other databases, such as Embase, PsycINFO, Web of Science, and others. If included, they might contribute to findings that would enrich our results and further increase the robustness of the present study. The same limitation pertains to the exclusion of articles written in languages other than English. Articles published in languages other than English were not included, and therefore certain information may be missed.

## Data Availability

Data sharing is not applicable.

## Supplementary Materials

The following supporting information can be downloaded at https://www.mdpi.com/article/10.3390/brainsci15101130/s1. Refs. [65,66,67,68,69,70] are cited in the Supplementary Materials file.

## Abbreviations

The following abbreviations are used in this manuscript:

CR	Cognitive remediation
