# Supplementary material for: Cognitive Remediation as a Tool for Enhancing Treatment Dimensions of Schizophrenic Symptomatology: A Systematic Review of Randomized Controlled Trials
Source: Brain Sci. 2025 Oct 21;15(10):1130. doi: 10.3390/brainsci15101130 (PMC12564651; doi:10.3390/brainsci15101130)
Supplement: Supplementary file 1 [file brainsci-15-01130-s001.zip › Supplementary Table S1.pdf]

Supplementary Table S1

Critical Appraisal Criteria (JBI Revised Checklist for RCTs updated tool)

Criteria	Addressed bias	Applied scale	Scale
1. Was true randomization used for assignment of participants to treatment groups?	Bias related to selection and allocation	1 - 4	1 [Yes]<br>2 [No]<br>3 [Unclear]<br>4 [Not Applicable]
2. Was allocation to treatment groups concealed?	Bias related to selection and allocation	1 - 4	1 [Yes]<br>2 [No]<br>3 [Unclear]<br>4 [Not Applicable]
3. Were treatment groups similar at baseline?	Bias related to selection and allocation	1 - 4	1 [Yes]<br>2 [No]<br>3 [Unclear]<br>4 [Not Applicable]
4. Were participants blind to treatment assignment?	Bias related to administration of intervention/exposure	1 - 4	1 [Yes]<br>2 [No]<br>3 [Unclear]<br>4 [Not Applicable]
5. Were those delivering treatment blind to treatment assignment?	Bias related to administration of intervention/exposure	1 - 4	1 [Yes]<br>2 [No]<br>3 [Unclear]<br>4 [Not Applicable]
6. Were treatment groups treated identically other than the intervention of interest?	Bias related to administration of intervention/exposure	1 - 4	1 [Yes]<br>2 [No]<br>3 [Unclear]<br>4 [Not Applicable]

7. Were outcome assessors blind to treatment assignments?	Bias related to assessment, detection and measurement of the outcome	1 - 4	1 [Yes]<br>2 [No]<br>3 [Unclear]<br>4 [Not Applicable]
8. Were outcomes measured in the same way for treatment groups?	Bias related to assessment, detection and measurement of the outcome	1 - 4	1 [Yes]<br>2 [No]<br>3 [Unclear]<br>4 [Not Applicable]
9. Were outcomes measured in a reliable way?	Bias related to assessment, detection and measurement of the outcome	1 - 4	1 [Yes]<br>2 [No]<br>3 [Unclear]<br>4 [Not Applicable]
10. Was follow up complete and if not, were differences between groups in terms of their follow up adequately described and analyzed?	Bias related to participant retention	1 - 4	1 [Yes]<br>2 [No]<br>3 [Unclear]<br>4 [Not Applicable]
11. Were participants analyzed in the groups to which they were randomized?	Statistical conclusion validity	1 - 4	1 [Yes]<br>2 [No]<br>3 [Unclear]<br>4 [Not Applicable]
12. Was appropriate statistical analysis used?	Statistical Conclusion Validity	1 - 4	1 [Yes]<br>2 [No]<br>3 [Unclear]<br>4 [Not Applicable]

13. Was the trial design appropriate and any deviations from the standard RCT design (individual randomization, parallel groups) accounted for in the conduct and analysis of the trial?	Statistical Conclusion Validity	1 - 4	1 [Yes]<br>2 [No]<br>3 [Unclear]<br>4 [Not Applicable]
