# Supplementary material for: Cognitive Remediation as a Tool for Enhancing Treatment Dimensions of Schizophrenic Symptomatology: A Systematic Review of Randomized Controlled Trials
Source: Brain Sci. 2025 Oct 21;15(10):1130. doi: 10.3390/brainsci15101130 (PMC12564651; doi:10.3390/brainsci15101130)
Supplement: Supplementary file 1 [file brainsci-15-01130-s001.zip › Supplementary Table S2.pdf]

Supplementary Table S2. Critical Appraisal of Studies Included

	Study	Randomization	Allocation	Similar treatment groups at baseline.	Participants blinded	Therapists blinded	Identical treatment of groups other than intervention	Assessors blinded	Identical way of measuring outcomes in groups	Reliability of measuring	Completeness of follow up/description of atrittion	Intention to Treat analysis	Appropirateness of Statistical analysis	Appropriateness of trial design	Risk of bias
5	Gharaeipour et al. 2012	unclear	No	Yes	No	Not applicable	Yes	Yes	Yes	Unclear	Yes	Yes	No	Yes	Moderate
26	Zhu, et al., 2021	Yes	Yes	Yes	Unclear	Not applicable	Yes	Yes	Yes	Unclear	Yes	No	Yes	Yes	Moderate
59	Vita et al., 2011b	Yes	Unclear	Yes	No	Not applicable	Yes	Yes	Yes	Yes	Yes	Yes	No	Yes	Moderate
92	Penadés et al., 2006	Yes	Yes	Yes	No	Not applicable	Yes	Yes	Yes	Unclear	Yes	Yes	Yes	Yes	Low
95	Vita et al., 2011a	Yes	No	Yes	No	Not applicable	Yes	Yes	Yes	Yes	Yes	Yes	Yes	Yes	Low
106	Zhu et al., 2022	Yes	Yes	Yes	No	Not applicable	Yes	Yes	Yes	Yes	Yes	Yes	Yes	Yes	Low
122	Zhu et al., 2020	Unclear	No	Yes	No	Not Applicable	Yes	Yes	Yes	Yes	Yes	Yes	Yes	Yes	Moderate
139	Tan et al., 2016	Yes	Yes	Yes	No	Not Applicable	Yes	Yes	Yes	No	Yes	Yes	Yes	Yes	Low
191	d'Amato, et al., 2011	No	No	Yes	No	Not Applicable	Yes	Yes	Yes	No	Unclear	Unclear	Unclear	Yes	High
210	Ricarte et al., 2012	Yes	No	Yes	No	Not Applicable	Yes	Yes	Yes	Yes	Yes	No	Yes	Yes	Moderate
212	Omiya et al., 2016	No	No	Yes	No	Not Applicable	Yes	No	Yes	Unclear	Yes	Yes	No	Yes	Low
219	Wykes et al., 2007	Yes	No	Yes	No	Not Applicable	Yes	Yes	Yes	Unclear	Yes	Yes	Yes	Yes	Moderate
225	Rakitzi et al., 2016	Yes	No	Yes	No	Not Applicable	Yes	Yes	Yes	No	Yes	Unclear	Yes	Yes	Moderate
226	Wykes et al., 2003	Yes	Yes	Yes	No	Not Applicable	Yes	Yes	Yes	No	Yes	Yes	No	Yes	Moderate

336	Sachs et al., 2012	No	No	Unclear	No	Not Applicable	Yes	No	Yes	No	Yes	No	No	Yes	High
393	Fathi et al., 2025	Yes	Yes	Yes	Yes	No Applicable	Yes	Yes	Yes	No	Yes	Yes	Yes	Yes	Low
402	Zhang et al., 2024	Yes	Yes	Yes	No	Not Applicable	Yes	No	Yes	No	Unclear	Unclear	Unclear	Unclear	High
408	Giuliani et al., 2024	No	No	No	No	Not Applicable	No	No	Yes	No	Unclear	Unclear	No	Yes	High
461	Li et al., 2022	Unclear	No	Yes	No	Not Applicable	Yes	Yes	Yes	No	Yes	No	Yes	Yes	Moderate
463	Dai et al., 2022	Yes	Unclear	Yes	No	Not Applicable	Yes	Yes	Yes	No	Yes	Yes	Yes	Yes	Moderate
471	Fekete et al., 2022	Yes	Unclear	Yes	No	Not Applicable	Yes	Yes	Yes	No	Yes	No	Yes	Yes	Moderate
488	Sampedro et al., 2021	Yes	Yes	Unclear	No	Not Applicable	Yes	Yes	Yes	Unclear	Yes	Yes	Yes	Yes	Moderate
499	Rocha et al., 2021	Yes	No	Unclear	No	Not Applicable	Yes	Yes	Yes	No	Yes	No	Yes	Yes	Moderate
511	Bossert et al., 2020	No	No	Yes	No	Not Applicable	Yes	No	Yes	No	Yes	Yes	Yes	Yes	Moderate
578	Matsuda et al., 2018	Yes	Yes	No	No	Not Applicable	Yes	Yes	Yes	No	Yes	No	Yes	Yes	Moderate
611	Peña et al., 2016	Yes	Unclear	Yes	No	Not Applicable	Yes	Yes	Yes	No	yes	no	yes	yes	Moderate
665	Cella et al., 2014	Yes	Yes	Yes	No	Not Applicable	Yes	No	Yes	Unclear	yes	no	yes	yes	Moderate
668	Sánchez et al., 2014	Yes	No	Unclear	No	No Applicable	Yes	Yes	Yes	Unclear	yes	no	yes	yes	Moderate
707	Klingberg et al., 2011	Yes	Yes	Yes	No	Not Applicable	Yes	Yes	Yes	Yes	Yes	Yes	Yes	Yes	Low
741	Kayser et al., 2006	Unclear	No	Yes	No	Not Applicable	Unclear	No	Yes	No	Yes	Yes	No	Unclear	High
755	Reeder et al., 2004	Unclear	No	Unclear	No	Not Applicable	Unclear	Yes	Yes	No	Unclear	No	No	Yes	High
815	Yamanushi et al., 2024	Yes	Unclear	Unclear	no	Not Applicable	Yes	Yes	Yes	No	Yes	Yes	Yes	Yes	Moderate
932	Tao et al., 2015	Unclear	No	Yes	No	Not Applicable	Yes	Unclear	Yes	no	Yes	No	No	Yes	High

965	Ojeda et al., 2012	No	No	Unclear	Yes	Not Applicable	Yes	Unclear	Yes	no	Yes	No	No	Yes	High
990	Beigi et al., 2008	Unclear	No	Unclear	No	Not Applicable	Yes	Yes	Yes	Unclear	yes	No	No	Yes	High
