# Supplementary material for: Cognitive Remediation as a Tool for Enhancing Treatment Dimensions of Schizophrenic Symptomatology: A Systematic Review of Randomized Controlled Trials
Source: Brain Sci. 2025 Oct 21;15(10):1130. doi: 10.3390/brainsci15101130 (PMC12564651; doi:10.3390/brainsci15101130)
Supplement: Supplementary file 1 [file brainsci-15-01130-s001.zip › Supplementary Table S3.pdf]

Table S3. Interpretation and Benchmarks for Common Effect Size Indices in Behavioral and Cognitive Neuroscience

Effect size	Typical use / model	Formula (conceptual)	Interpretation benchmarks	Key references
**η^2 (Eta squared)**	One-way ANOVA / MANOVA	η^2 = SS_effect / SS_total	.01 = small .06 = medium .14 = large	Cohen (1988); Richardson (2011)
**ηp^2 (np^2)** Partial eta squared	Mixed / repeated-measures ANOVA	ηp^2 = SS_effect / (SS_effect + SS_error)	.01 = small .06 = medium .14 = large	Richardson (2011); Lakens (2013)
**Cohen's d**	t-tests / pairwise comparisons	$d = (M_1 – M_2)$ / SD_pooled	.20 = small .50 = medium .80 = large	Cohen (1988); Sawilowsky (2009)
**r / β ("total b")**	Correlation / regression / SEM	$r = \sqrt{(\eta^2)}$ or $r = d / \sqrt{(d^2 + 4)}$	.10 = small .30 = medium .50 = large	Cohen (1992); Rosenthal (1994)

Conversion formulas:
$r = d / \sqrt{(d^2 + 4)}$ | $d = 2r / \sqrt{(1 - r^2)}$ | $f = \sqrt{(\eta p^2 / (1 - \eta p^2))}$

References

Cohen J. (1988). *Statistical Power Analysis for the Behavioral Sciences* (2nd ed.). Erlbaum.
Cohen J. (1992). A power primer. *Psychological Bulletin, 112*(1), 155–159.
Richardson J. T. E. (2011). Eta squared and partial eta squared as measures of effect size in educational research. *Educational Research Review, 6*(2), 135–147.
Lakens D. (2013). Calculating and reporting effect sizes to facilitate cumulative science. *Frontiers in Psychology, 4*, 863.
Sawilowsky S. S. (2009). New effect size rules of thumb. *Journal of Modern Applied Statistical Methods, 8*(2), 467–474.
Rosenthal R. (1994). Parametric measures of effect size. In H. Cooper & L. V. Hedges (Eds.), *The Handbook of Research Synthesis* (pp. 231–244). Russell Sage Foundation.
