# Supplementary material for: Cognitive Remediation as a Tool for Enhancing Treatment Dimensions of Schizophrenic Symptomatology: A Systematic Review of Randomized Controlled Trials
Source: Brain Sci. 2025 Oct 21;15(10):1130. doi: 10.3390/brainsci15101130 (PMC12564651; doi:10.3390/brainsci15101130)
Supplement: Supplementary file 1 [file brainsci-15-01130-s001.zip › Supplementary Table S4.pdf]

Study	True Randomization	Allocation to treatment groups concealed?	Groups similar at baseline?	Participants blind to treatment assignment?	Therapists delivering treatment blind to treatment assignment?	Treatment groups treated identically other than intervention of interest?	Outcome assessors blind to treatment assignments?	Outcomes measured in the same way for treatment groups?	Outcomes measured in a reliable way?	Was follow up complete or, if not, adequately described?	Participants analyzed in the groups to which they were randomized?	Appropriate statistical analysis used?	Was the trial design appropriate?
**Gharaeipour & Scott, 2012**	Unclear	No	Yes	Unclear	N/A	Yes	Yes	Yes	Unclear	Yes	Yes	No	Yes
**Zhu et al., 2021**	Yes	Yes	Yes	Unclear	N/A	Yes	Yes	Yes	Unclear	Yes	No	Yes	Yes
**Vita et al., 2011b**	Yes	Unclear	Yes	No	N/A	Yes	Yes	Yes	Yes	Yes	Yes	No	Yes
**Penadés et al., 2006**	Yes	Yes	Yes	No	N/A	Yes	Yes	Yes	Unclear	Yes	Yes	Yes	Yes
**Vita et al., 2011a**	Yes	No	Yes	No	N/A	Yes	Yes	Yes	Yes	Yes	Yes	Yes	Yes
**Zhu et al., 2022**	Yes	Yes	Yes	No	N/A	Yes	Yes	Yes	Yes	Yes	Yes	Yes	Yes
**Zhu et al., 2020**	Unclear	No	Yes	No	N/A	Yes	Yes	Yes	Yes	Yes	Yes	Yes	Yes
**Tan et al., 2016**	Yes	Yes	Yes	No	N/A	Yes	Yes	Yes	No	Yes	Yes	Yes	Yes
**D’Amato et al., 2011**	No	No	Yes	No	N/A	Yes	Yes	Yes	No	Unclear	Unclear	Unclear	Yes
**Ricarte et al., 2012**	Yes	No	Yes	No	N/A	Yes	Yes	Yes	Yes	Yes	No	Yes	Yes

Study	True Randomization	Allocation to treatment groups concealed?	Groups similar at baseline?	Participants blind to treatment assignment?	Therapists delivering treatment blind to treatment assignment?	Treatment groups treated identically other than intervention of interest?	Outcome assessors blind to treatment assignments ?	Outcomes measured in the same way for treatment groups?	Outcomes measured in a reliable way?	Was follow up complete or, if not, adequately described?	Particip ants analyzed in the groups to which they were randomi zed?	Appropri ate statistical analysis used?	Was the trial design approp riate?
**Omiya et al., 2016**	No	No	Yes	No	N/A	Yes	No	Yes	Unclear	Yes	Yes	No	Yes
**Wykes et al., 2007**	Yes	No	Yes	No	N/A	Yes	Yes	Yes	Unclear	Yes	Yes	Yes	Yes
**Rakitzi et al., 2016**	Yes	No	Yes	No	N/A	Yes	Yes	Yes	No	Yes	Unclear	Yes	Yes
**Wykes et al., 2003**	Yes	Yes	Yes	No	N/A	Yes	Yes	Yes	No	Yes	Yes	No	Yes
**Sachs et al., 2012**	No	No	Unclear	No	N/A	Yes	No	Yes	No	Yes	No	No	Yes
**Fathi Azar et al., 2025**	Yes	Yes	Yes	Yes	N/A	Yes	Yes	Yes	No	Yes	Yes	Yes	Yes
**Zhang et al., 2024**	Yes	Yes	Yes	No	N/A	Yes	No	Yes	No	Unclear	Unclear	Unclear	Yes
**Giuliani et al., 2024**	Unclear	No	No	No	N/A	No	No	Yes	No	Unclear	Unclear	No	Yes
**Li et al., 2022**	Unclear	No	Yes	No	N/A	Yes	Yes	Yes	No	Yes	No	Yes	Yes
**Dai et al., 2022**	Yes	Unclear	Yes	No	N/A	Yes	Yes	Yes	No	Yes	Yes	Yes	Yes

Study	True Randomization	Allocation to treatment groups concealed?	Groups similar at baseline?	Participants blind to treatment assignment?	Therapists delivering treatment blind to treatment assignment?	Treatment groups treated identically other than intervention of interest?	Outcome assessors blind to treatment assignments?	Outcomes measured in the same way for treatment groups?	Outcomes measured in a reliable way?	Was follow up complete or, if not, adequately described?	Participants analyzed in the groups they were randomized?	Appropriate statistical analysis used?	Was the trial design appropriate?
**Fekete et al., 2022**	Yes	Unclear	Yes	No	N/A	Yes	Yes	Yes	No	Yes	No	Yes	Yes
**Sampedro et al., 2021**	Yes	Yes	Unclear	No	N/A	Yes	Yes	Yes	Unclear	Yes	Yes	Yes	Yes
**Rocha et al., 2021**	Yes	No	Unclear	No	N/A	Yes	Yes	Yes	No	Yes	No	Yes	Yes
**Bossert et al., 2020**	No	No	Yes	No	N/A	Yes	No	Yes	No	Yes	Yes	Yes	Yes
**Matsuda et al., 2018**	Yes	Yes	No	No	N/A	Yes	Yes	Yes	No	Yes	Yes	No	Yes
**Peña et al., 2016**	Yes	Unclear	Yes	No	N/A	Yes	Yes	Yes	No	Yes	No	Yes	Yes
**Cella et al., 2014**	Yes	Yes	Yes	No	N/A	Yes	No	Yes	Unclear	Yes	No	Yes	Yes
**Sánchez et al., 2014**	Yes	No	Unclear	No	N/A	Yes	Yes	Yes	Unclear	Yes	No	Yes	Yes
**Klingberg et al., 2011**	Yes	Yes	Yes	No	N/A	Yes	Yes	Yes	Yes	Yes	Yes	Yes	Yes
**Kayser et al., 2006**	Unclear	No	Yes	No	N/A	Unclear	No	Yes	No	Yes	Yes	No	Unclear

Study	True Randomization	Allocation to treatment groups concealed?	Groups similar at baseline?	Participants blind to treatment assignment?	Therapists delivering treatment blind to treatment assignment?	Treatment groups treated identically other than intervention of interest?	Outcome assessors blind to treatment assignments?	Outcomes measured in the same way for treatment groups?	Outcomes measured in a reliable way?	Was follow up complete or, if not, adequately described?	Participants analyzed in the groups to which they were randomized?	Appropriate statistical analysis used?	Was the trial design appropriate?
**Reeder et al., 2004**	Unclear	No	Unclear	No	N/A	Unclear	Yes	Yes	No	Unclear	Yes	Yes	No
**Yamanushi et al., 2024**	Yes	Unclear	Unclear	No	N/A	Yes	Yes	Yes	No	Yes	Yes	Yes	Yes
**Tao et al., 2015**	Unclear	No	Yes	No	N/A	Yes	Unclear	Yes	No	Yes	No	No	Yes
**Ojeda et al., 2012**	No	No	Unclear	Yes	N/A	Yes	Unclear	Yes	No	Yes	No	No	Yes
**Beigi et al., 2008**	Unclear	No	Unclear	No	N/A	Yes	Yes	Yes	Unclear	Yes	No	No	Yes
